# The complete mitochondrial genome of the Chinese *Daphnia pulex* (Cladocera, Daphniidae)

**DOI:** 10.3897/zookeys.615.8581

**Published:** 2016-09-07

**Authors:** Xuexia Geng, Ruixue Cheng, Tianyi Xiang, Bin Deng, Yaling Wang, Daogui Deng, Haijun Zhang

**Affiliations:** 1 College of Life Science, Huaibei Normal University, Huaibei 235000, China; 2 No.1 High School of Huaibei Anhui, Huaibei 235000, China

**Keywords:** Daphnia pulex, gene order, mitochondrial genome, secondary structure

## Abstract

*Daphnia pulex* has played an important role in fresh-water ecosystems. In this study, the complete mitochondrial genome of *Daphnia pulex* from Chaohu, China was sequenced for the first time. It was accomplished using long-PCR methods and a primer-walking sequencing strategy with genus-specific primers. The mitogenome was found to be 15,306 bp in length. It contained 13 protein-coding genes, two rRNA genes, 22 tRNA genes and a typical control region. This research revealed an overall A+T content of 64.50%. All of the 22 typical animal tRNA genes had a classical clover-leaf structure except for *trnS1*, in which its DHU arm simply formed a loop. The lengths of small and large rRNA were 744 bp and 1,313 bp, respectively. The A+T-rich region was 723 bp in length, which is longer than that from the North American species (689 bp). In terms of structure and composition, many similarities were found between the Chinese and North American *Daphnia pulex*.

## Introduction

Cladocerans (“water fleas”) are an important component of the microcrustacean zooplankton. Their habitats are mostly continental fresh and saline waters (Forró et al. 2008). *Daphnia pulex* has become a well-known model species for studying evolutionary biology, environmental biology and ecology (Miner et al. 2013, Geng et al. 2016). Although other related research has been done (Roland et al. 2011, Geng et al. 2014), there are still some difficulties with species identification. In this study, meaningful data to assist in the taxonomy of different species of *Daphnia* is provided, and variations in similar morphological groups using molecular tools are analysed (Petrusek et al. 2012).

The sequence and structure of mitochondrial genomes has been frequently used to study phylogenetic relationships of animal taxa. More specifically, the unusual characters of mitochondrial genome DNA, for instance its small size, fast evolutionary rate, simple structure, maternal inheritance and high informational content, have been widely regarded as a molecular marker for phylogenetic analysis (Wilson et al. 2000, Chao et al. 2014, Ma et al. 2015).

All metazoan animals contain their own circular mitochondrial genome with two strands (a J-strand and an N-strand) (Simon et al. 2014), which range from 14 kb to 42 kb in length (Wolstenholme 1992). These typically encoded 37 genes, namely: 2 rRNA genes (*16S rRNA* and *12S rRNA*), 22 tRNA genes, and 13 protein-coding genes (*COI*, *COII*, *COIII*, *Cytb*, *ATP6*, *ATP8*, *ND1*, *ND2*, *ND3*, *ND4*, *ND4L*, *ND5*, *ND6*) (Boore 1999). Moreover, the non-coding region (also called the control region or D-loop), which with significant functions in the regulation and initiation of mitochondrial DNA transcription and replication (Brown et al. 1979, Shadel and Clayton 1993, Zhang and Hewitt 1997). Complete mitochondrial genome sequences are more informative than shorter sequences of individual genes but also provide a set of genomic characters. This led to the recognition of relative positions of different genes, RNA secondary structures and modes of control of replication and transcription (Masta and Boore 2008). However, the complete mitochondrial genome sequences data on *Daphnia* released in Genbank is far from enough.

The main purpose of this study was to disclose the complete mitochondrial genome sequence of the Chinese *Daphnia pulex* for the first time, and to compare its features with other available cladoceran mitochondrial genomes.

This study also served as a useful source of information for both nuclear and mitochondrial markers in comparative analyses of the evolution of mitochondrial genomes in Cladocerans.

## Materials and methods

### Samples and DNA extraction

Total DNA was extracted from individual specimens using a TIANamp Micro DNA Kit (TIANGEN BIOTECH (BEIJING) CO., LTD) following manufacturer protocols. DNA samples were stored at -20 °C until further use.

### PCR amplifications and sequencing

The *Daphnia pulex* mitochondrial genome was amplified using five pairs of primers (Table 1). To obtain the complete sequences of Chinese *Daphnia pulex*, short-PCR and long-PCR methods were used. The primers employed in this study were designed based on the mitochondrial genomes of the North American *Daphnia pulex* (GenBank accession number AF117817) (Crease 1999) by using an NCBI primer-BLAST (http://www.ncbi.nlm.nih.gov/tools/primer-blast/).

**Table 1.** Details of the primers used to amplify the mitogenome of Chinese *Daphnia pulex*.

Primer pair	Size (bp)	Primer sequence(5’-3’)
F1		AGAAGGGAATTTGAGCTCTTTTWGT
R1	5450	TTACCCTAGGGATAACAGCGTAA
F2		TCGTCTCGTCATTCATACCAGC
R2	2221	GTGCCAGCAGYYGCGGTTANAC
F3		ATAAYAGGGTATCTAATCCTRGT
R3	3122	ACTTCCWGATTGTCCYAAYTC
F4		ACTACCCGCAAACGATCTGG
R4	4000	TGGGATGGGTTGGGGCTAAT
F5		AGCCCCAAAAATTGGATTTCCC
R5	750	TGGCTTCGGCAACGGATAG

The PCRs were performed by using an Eppendorf Thermal Cycler (5331AH760577, Eppendorf, Germany) with a 25 µL volume reaction mixture containing 2.5 µL 10×LA-Taq Buffer II(Mg^2+^ plus), 4 µL dNTP Mixture (2.5 mM), 2 µL DMSO, 1 µL genomic DNA, 1 µL 10 µM of each primer, 0.5 µL MgCl_2_ (25 mM) and 0.25 µL 2.5 units of LA Taq polymerase (TaKaRa Biomedical, Japan), and 12.25 µL distilled water.

The reaction conditions were one cycle of denaturation at 95 °C 5 min, 35 cycles of denaturation at 95 °C 30 s, annealing at 50 °C 30 s, extension at 72 °C for 2 to 8 min and a final extension at 72 °C for 10 min. Each amplicon (5 µL) was examined with agarose gel electrophoresis to validate amplification efficiency. PCR products were sequenced directly by primer walking from both directions after purification.

### Analysis and annotation

The raw sequences of mitochondrial genome were edited and assembled by using the program Seqman (DNAStar, Inc.) and then adjusting them manually. Protein-coding genes and rRNA genes were identified by the MITOS WebServer (http://mitos.bioinf.uni-leipzig.de/index.py) and the similarity between *Daphnia pulex* and that published in NCBI database were distinguished by BLAST search function (http://www.ncbi.nlm.nih.gov/BLAST/). Nucleotide sequences of PCGs were translated using the invertebrate mitochondrial genetic code. The tRNA genes were initially identified by the MITOS WebServer (http://mitos.bioinf.uni-leipzig.de/index.py) and their secondary structures were predicted and modified based on other metazoan’s secondary structure of tRNA genes.

The exact initiation and termination codons were identified by using Clustal X version 2.0 (Larkin et al. 2007) and relied on reference sequences from other invertebrates. Nucleotide composition and codon usage were calculated with MEGA 6.0 software (Tamura et al. 2013). The sequence data has been deposited into GenBank database under the accession number KT003819.

## Results and discussion

### Genome organization and base composition

**Figure 1.**
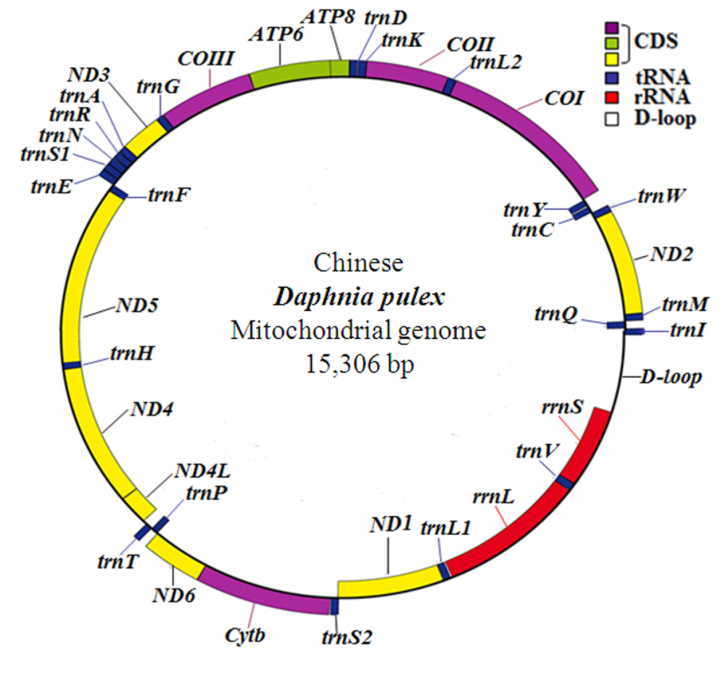
Structure of Chinese *Daphnia pulex* mitochondrial genome. *COI*, *COII*, *COIII* refer to the cytochrome oxidase subunits, *Cytb* refers to cytochrome b, *ND1* - *ND6* refer to NADH dehydrogenase components, and *rrL* and *rrnS* refer to rRNAs. tRNA genes are denoted by one letter symbol according to the IPUC-IUB single-letter amino acid codes. L1, L2, S1 and S2 denote tRNALeu(CUN), tRNALeu(UUR), tRNASer(AGN) and tRNASer(UCN), respectively. D-loop indicates A+T-rich region. Gene names outside the ring are coded on the majority strand while those inside are on the minority strand.

The mitochondrial genomes of the Chinese *Daphnia pulex* used in this study were similar to that of the *Daphnia pulex* in North America (Crease 1999). The complete mitochondrial genome of Chinese *Daphnia pulex* was a circular molecule 15,306 bp in size, containing 13 protein-coding genes, 22 tRNA genes, 2 rRNA genes for both the small and large subunits (*rrnS* and *rrnL*) and a putative control region (Fig. 1). Among all the 37 genes, 23 genes were encoded on the J-strand. The remaining genes were encoded on the N-strand. 8 overlaps were found between adjacent genes (29 bp in total), among which the longest was 10 bp located at *trnS2* and *ND1*. This included 15 intergenic spacers that ranged from 1 to 31 bp (84 bp in total), of which only one spacer was longer than 10 bp. That occurred between *ND4L* and *trnT*.

The mitochondrial genome of the Chinese *Daphnia pulex* has an A+T content of 64.50%, which is a little higher than that of the North American species (62.26%). Furthermore, it was determined that the AT skew was 0.006, and the GC skew was -0.107. AT skew and GC skew for a given strand were calculated as (G-C)/(G+C) and (A-T)/(A+T), respectively, with negative values in skewness meaning the coding strand is enriched for T or C. In contrast, positive values infer more As and Gs. On the whole, AT skew was slightly negative, or positive in the third codon position of vestimetiferans, and GC skew was more negative than AT skew (Table 2). Nucleotide bias can also be reflected by codon usage. We found that the RSCU (Relative Synonymous Codon Usage) value of NNA and NNU codons were greater than 1, which indicates that codons were biased in favor of codons with A or T in the third position (Table 3).

**Table 2.** Nucleptide composition in different regions of the *Daphnia pulex* mitochondrial from different areas.

	areas	length (bp)	A(%)	T(%)	G(%)	C(%)	A+T(%)	G+C(%)	AT skew	GC skew
The whole mitochondrial genome	Ch	15306	32.45	32.04	15.84	19.66	64.49	35.50	0.006	-0.107
	Na	15333	31.47	30.79	16.69	21.05	62.26	37.74	0.011	-0.116
Protein-coding genes	Ch	11026	24.73	38.64	18.31	18.32	63.37	36.63	-0.219	-0.0002
	Na	11074	23.39	37.04	19.40	20.17	60.43	39.57	-0.226	-0.019
1st	Ch	3665	26.63	29.66	25.21	18.50	56.29	43.71	-0.054	0.154
	Na	3681	25.94	29.34	25.42	19.29	55.28	44.71	-0.061	0.137
2nd	Ch	3665	17.11	45.70	16.92	20.27	62.81	37.19	-0.455	-0.090
	Na	3681	17.33	45.18	16.54	20.95	62.51	37.49	-0.445	-0.117
3rd	Ch	3665	30.23	40.52	12.91	16.34	70.75	29.25	-0.145	-0.118
	Na	3681	26.68	36.57	16.30	20.46	63.25	36.76	-0.156	-0.113
tRNA	Ch	1448	33.22	33.01	18.99	14.78	66.23	33.77	0.003	0.124
	Na	1452	32.78	32.99	19.42	14.81	65.77	34.23	-0.003	0.134
rRNA	Ch	2057	34.03	34.71	16.67	14.58	68.74	31.25	-0.010	0.067
	Na	2067	35.41	32.41	15.19	16.98	67.82	32.17	0.044	-0.056
D-loop	Ch	723	32.09	33.33	16.04	18.53	65.42	34.57	-0.019	-0.072
	Na	689	32.37	34.69	15.38	17.56	67.06	32.94	-0.035	-0.066

AT skew = (A-T)/(A+T), GC skew = (G-C)/(G+C).

**Table 3.** Codon usage of the Chinese *Daphnia pulex* mitogenome.

Codon	Count	RSCU	Codon	Count	RSCU	Codon	Count	RSCU	Codon	Count	RSCU
UUU(F)	20.1	1.39	UCU(S)	8	2.04	UAU(Y)	7.9	1.27	UGU(C)	3.5	1.3
UUC(F)	8.8	0.61	UCC(S)	3	0.76	UAC(Y)	4.5	0.73	UGC(C)	1.9	0.7
UUA(L)	12.3	1.76	UCA(S)	3.7	0.94	UAA(*)	5.9	1.01	UGA(*)	4.5	0.77
UUG(L)	6.3	0.9	UCG(S)	1.8	0.45	UAG(*)	7.2	1.22	UGG(W)	3.8	1
CUU(L)	9.2	1.32	CCU(P)	5	1.71	CAU(H)	3.3	1.23	CGU(R)	1.2	0.63
CUC(L)	5.2	0.74	CCC(P)	3.2	1.08	CAC(H)	2.1	0.77	CGC(R)	1.1	0.59
CUA(L)	5.3	0.76	CCA(P)	1.5	0.5	CAA(Q)	3.5	1.08	CGA(R)	1.9	1.05
CUG(L)	3.6	0.52	CCG(P)	2.1	0.71	CAG(Q)	2.9	0.92	CGG(R)	1	0.55
AUU(I)	12.2	1.57	ACU(T)	6	1.88	AAU(N)	5.6	1.4	AGU(S)	4.6	1.18
AUC(I)	5	0.64	ACC(T)	2.4	0.75	AAC(N)	2.4	0.6	AGC(S)	2.5	0.63
AUA(I)	6.2	0.79	ACA(T)	2.9	0.92	AAA(K)	4.4	1.1	AGA(R)	3.8	2.06
AUG(M)	5	1	ACG(T)	1.5	0.46	AAG(K)	3.6	0.9	AGG(R)	2.1	1.13
GUU(V)	5.2	1.41	GCU(A)	4	1.81	GAU(D)	4.3	1.23	GGU(G)	2.4	0.62
GUC(V)	2.3	0.62	GCC(A)	1.8	0.83	GAC(D)	2.7	0.77	GGC(G)	2.1	0.54
GUA(V)	4.6	1.24	GCA(A)	2.2	0.97	GAA(E)	1.8	0.69	GGA(G)	4.4	1.13
GUG(V)	2.7	0.73	GCG(A)	0.8	0.38	GAG(E)	3.4	1.31	GGG(G)	6.6	1.71

RSCU: Relative Synonymous Codon Usage.

Amino acids are denoted as one-letter symbol according to the IUPAC-IUB single letter amino acid codes.

### Protein-coding genes

The complete mitochondrial DNA of Chinese *Daphnia pulex* from Chaohu had 13 protein-coding genes. Nine of these genes were located on the J-strand while the others were found on the N-strand; the same as the *Daphnia pulex* in North America (Table 3). Ten out of these 13 protein-coding genes initiated with typical ATN codons. *ND2*, *COII*, *ATP6*, *COIII*, *ND4*, *Cytb* and *ND1* started with ATG, *COI* initiated with ATA, and moreover *ND3* and *ND6* used ATC as the initiating codon. The *ATP8* and *ND5* genes used GTG. The *ND4L* gene used none of these as initiating codon, but GCT.

As is the case with some other arthropod species, the initiation functions of the *COI*codon has not been fully investigated. Atypical initiating codons for the *COI* gene in mitochondrial genomes have been reported in many studies, examples of these genes are: CGA (Gong et al. 2012), GTG (He et al. 2011), TTG (Hu et al. 2010, Li et al. 2012), ACG (Wilson et al. 2000), CCG (Fenn et al. 2007), ACC (Yamauchi et al. 2004), and TTA (Yamauchi et al. 2002). In *Drosophila*, *Locusta* and *Daphnia*, there are occasionally some uncommon quadruplets like, ATAA or ATTA, that may serve as an initiation codon (Wilson et al. 2000). One example of this is the *COI* gene in the North American *Daphnia pulex* initiating with ATTA (Crease 1999). However, the *COI* gene of Chinese *Daphnia pulex* started with classical ATA.

Nine of the 13 protein-coding genes used the typical termination codon TAN. *ND2* and *ATP8* terminated with TAG. *COIII*, *ND3*, *Cytb*, *ATP6*, *ND4L*, *ND6* and *ND1* all terminated with TAA. *COI*, *COII*, *ND4* and *ND5* used the incomplete termination codon T. Both of the complete termination codons TAG and TAA and two additional abbreviated termination codons T and TA were found in the North American *Daphnia pulex* (Table 4).

**Table 4.** Organization of the mitochondrial genomes of *Daphnia pulex* from Chinese Chaohu (Ch) and that from North America (Na).

Gene/strand	position		length		Start/stop codon	
	Ch	Na	Ch	Na	Start codon (Ch/Na)	Stop codon (Ch/Na)
trnI/J	1–64	1–64	64	64		
trnQ/N	66–133	66–133	68	68		
trnM/J	134–197	134–197	64	64		
ND2/J	198–1139	198–1185	942	988	ATG/ATG	TAG/T__
trnW/J	1138–1202	1186–1251	65	66		
trnC/N	1206–1268	1253–1316	63	64		
trnY/N	1278–1340	1328–1391	63	64		
COI/J	1350–2886	1397–2934	1537	1538	ATA/(A)TTA	T__/T__
trnL2/J	2887–2954	2935–3002	68	68		
COII/J	2956–3634	3004–3682	679	679	ATG/ATG	T__/T__
trnK/J	3635–3704	3683–3752	70	70		
trnD/J	3709–3773	3757–3821	65	65		
ATP8/J	3774–3935	3821–3982	162	162	GTG/GTG	TAG/TAG
ATP6/J	3929–4603	3976–4649	675	674	ATG/ATG	TAA/TA_
COIII/J	4603–5391	4650–5438	786	789	ATG/ATG	TAA/TAA
trnG/J	5393–5456	5439–5499	64	61		
ND3/J	5457–5810	5500–5852	354	353	ATC/ATT	TAA/TA_
trnA/J	5811–5874	5853–5918	64	66		
trnR/J	5876–5940	5920–5984	65	65		
trnN/J	5943–6010	5985–6051	68	67		
trnS1/J	6011–6075	6052–6116	65	65		
trnE/J	6076–6141	6117–6184	66	68		
trnF/N	6141–6205	6184–6249	65	66		
ND5/N	6207–7913	6250–7957	1707	1708	GTG/ATG	T__/T__
trnH/N	7908–7971	7952–8015	64	64		
ND4/N	7972–9292	8016–9336	1321	1321	ATG/ATG	T__/T__
ND4L/N	9295–9570	9339–9614	276	276	GCT/ATT	TAA/TAA
trnT/J	9602–9664	9646–9710	63	65		
trnP/N	9665–9730	9711–9775	66	65		
ND6/J	9733–10245	9778–10290	513	513	ATC/ATT	TAA/TAA
Cytb/J	10245–11378	10298–11431	1134	1134	ATG/ATG	TAA/TAA
trnS2/J	11379–11447	11432–11500	69	69		
ND1/N	11438–12373	11494–12426	936	936	ATG/ATG	TAA/TAA
trnL1/N	12377–12443	12430–12496	67	67		
rrnL/N	12454–13766	12506–13819	1313	1314		
trnV/N	13769–13840	13821–13892	72	72		
rrnS/N	13840–14583	13892–14644	744	753		
D-loop/J	14584–15306	14645–15333	723	689		

Note: J and N refer to the majority and minority strand, respectively. Position numbers refer to positions on the majority strand.

The use of incomplete termination codons on these genes might serve the purpose of avoiding overlapping nucleotides between adjacent genes (He et al. 2012). The incomplete termination codons would become functional termination codons after polycistronic transcript cleavage and polyadenylation processes have occured (Ojala et al. 1981). These incomplete codons and this mechanism has been commonly found in metazoan mitochondrial genomes (Wei et al. 2009, Liao et al. 2010). The total length of the 13 protein-coding genes was found to be 11,026 bp for the Chinese *Daphnia pulex*, which accounts for 63.37% of the total mitogenome length.

**Figure 2.**
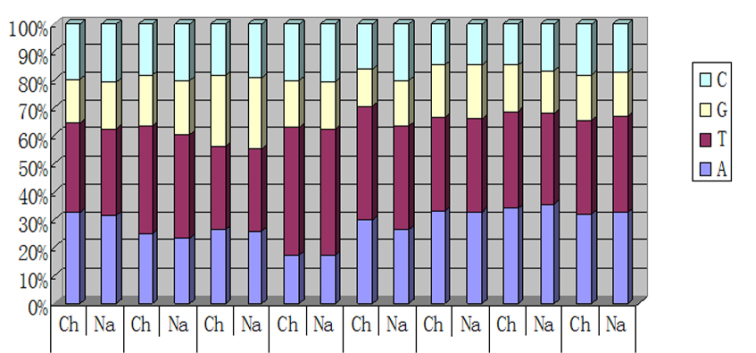
Nucleotide compositions of the two *Daphnia pulex* from Chinese Chaohu (Ch) and North America (Na). CDS: protein-coding genes; 1st: first codon position; 2nd: second codon position; 3rd: third codon position; tRNA: tRNA genes; rRNA: rRNA genes; D-loop: A+T-rich region. In addition, stop codons were excluded.

Many composition similarities were noted between the two different species compared in this study (Fig. 2).

### tRNA genes

**Figure 3.**
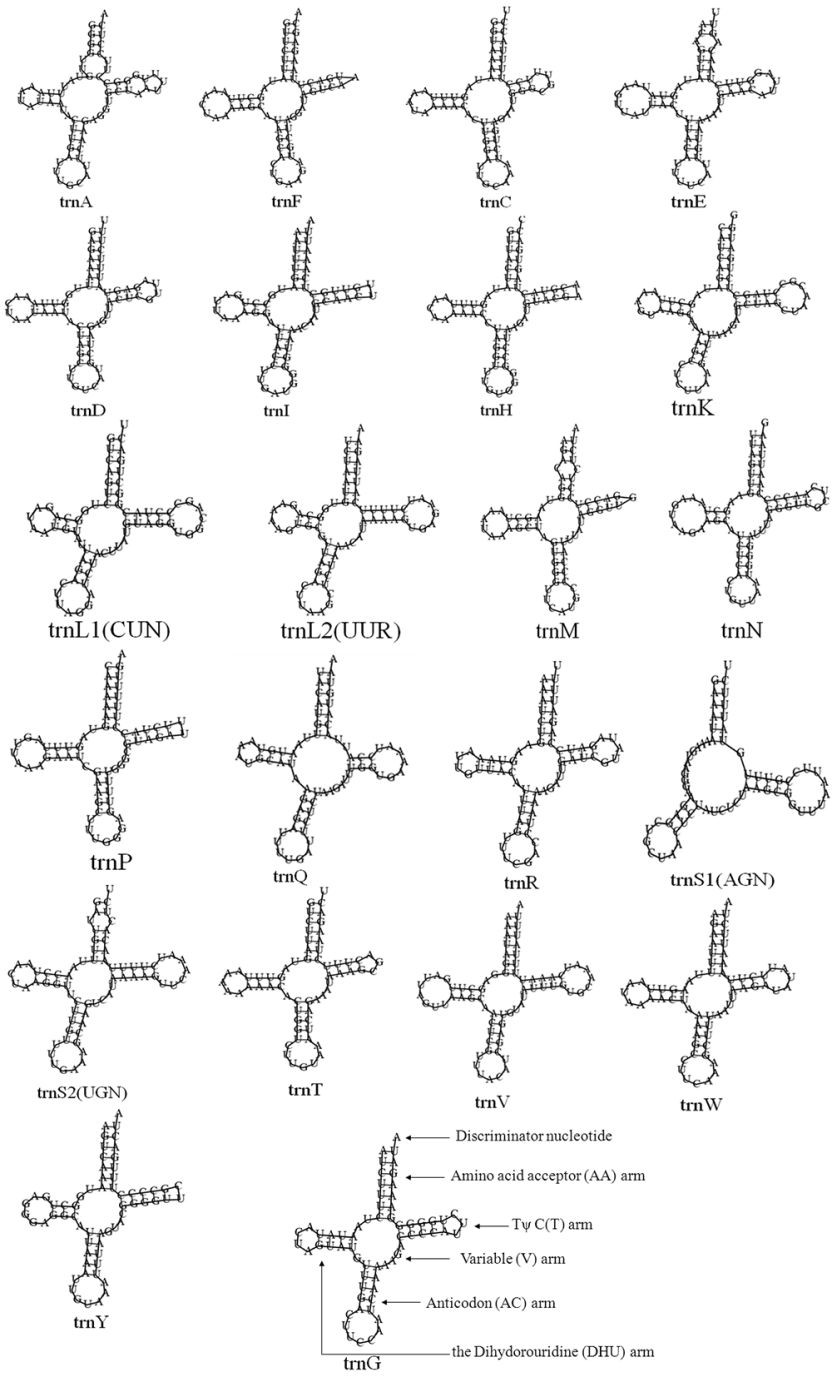
Inferred secondary structure of 22 tRNA genes in Chinese *Daphnia pulex* mtDNA genome.

All of the 22 typical arthropod tRNAs were found in the Chinese *Daphnia pulex* mitochondrial genome. They ranged from 63 to 72 bp in size. A schematic drawing of their respective secondary structures is shown in Figure 3. All tRNA genes had a clover-leaf structure except for *trnS1*, in which its DHU arm simply formed a loop. This loop in *trnS* is not uncommon in metazoan mitochondrial genomes (Crease and Little 1997). Whether or not the aberrant tRNAs lose their respective functions is still unknown. However, it’s possible this anomaly may be rectified by subsequent RNA-editing mechanisms (Lavrov et al. 2000, Masta and Boore 2004, Li et al. 2012).

Non-canonical pairs, which possessed non Watson-Crick matches, commonly manifest in mitochondrial tRNA gene secondary structures. There are 30 base pair mismatches present in the tRNA secondary structures of Chinese *Daphnia pulex* mtDNA, including 15 wobble G-U pairs, 13 U-G pairs , two U-U pairs, one A-A pair and one U-C pair mismatch (Fig. 3). Nevertheless, the post-transcriptional RNA-editing mechanism can rectify these mismatches to maintain tRNA functions (Tomita et al. 2001, Wang et al. 2014).

### rRNA genes

Both the *rrnL* and *rrnS* genes were present in Chinese *Daphnia pulex* mitochondrial genome. They were located between *trnL1* and the non-coding putative control region and separated by *trnV*, as similarly found in vertebrate mitochondrial genomes (Delisle and Strobeck 2002, Hwang et al. 2008, Chao et al. 2014).

Large and small ribosomal RNA genes (*rrnL* and *rrnS*) in Chinese *Daphnia pulex* were 1,313 bp and 744 bp long, respectively. The lengths of the two rRNAs were almost similar to that of the *Daphnia pulex* in North America (1,314 bp and 753 bp, respectively).

### Non-coding sequence

There are 15 non-coding regions ranging from 1 to 31 bp except for the A+T-rich region in the Chinese *Daphnia pulex* mitochondrial genome.

A 31 bp intergenic sequence was present between *ND4L* and *trnT*, which is also found in the North American *Daphnia pulex* mitochondrial DNA. The longest intergenic region in Chinese *Daphnia pulex* was the A+T-rich region. It was between *rrnS* and *trnI* with the length of 723 bp. It has an A+T content of 65.42%. It was a little longer than that of the North American *Daphnia pulex* mitochondrial DNA (689 bp), but lower in A+T content. This region usually contains replication and transcription areas in both vertebrates and invertebrates (Zhang and Hewitt 1997, Boore 1999). The stem-loop structure and the quantity of multiple repeats of AT sequences are notable features of the control region, ranging from 200 bp to 1,300 bp, and determine the difference in arthropod mitochondrial DNA size (Boore 1999).

### Phylogenetic analyses

**Figure 4.**
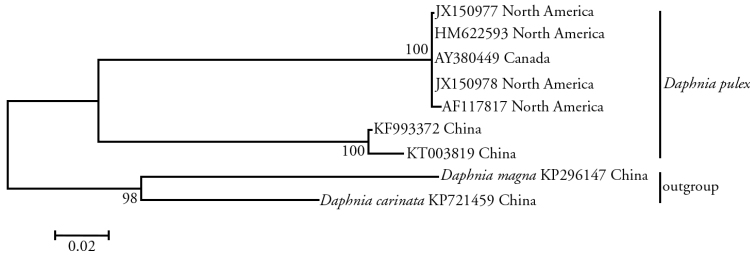
Phylogenetic tree obtained by the maximum-likelihood (ML) method and bootstrap values (1000 repetitions) of the branches were indicated. *Daphnia magna* and *Daphnia carinata* were used as outgroups.

The phylogenetic relationships among the *Daphnia pulex* from different areas were reconstructed based on nucleotide sequences of the *COI* gene by using the maximum likelihood (ML) mothod (Fig. 4). The phylogenetic analyses show that the Chinese and North American *Daphnia pulex* are recovered as two monophyletic clades with strong bootstrap support values (bs=100). They maybe evolved into two different species.

## Conclusion

The mapping of the mitochondrial genome of the Chinese *Daphnia pulex* was completed in this study. It was found to be 15,306 bp in length and had a similar composition in size and structure to the *Daphnia pulex* mitochondrial DNA in North America published in GenBank AF117817 (Crease 1999). However, the phylogenetic analysis showed that the Chinese and North American *Daphnia pulex* maybe evolved into two different species (Fig. 4). The complete mitogenome of the Chinese *Daphnia pulex* reported here is expected to supply more molecular information for further studies of the *Daphnia* phylogeny and for analyses on the taxonomic status of the Cladocera.
